# Hepatitis E Virus Infection in Vietnamese Pregnant Women with Hepatitis B: Prevalence and Clinical Outcomes

**DOI:** 10.1093/ofid/ofaf081

**Published:** 2025-02-10

**Authors:** Le Thi Hong Van, Hoang Van Tong, Bui Thanh Thuyet, Bui Lan Anh, Le Chi Cao, Do Thu Trang, Nghiem Xuan Hoan, Trinh Xuan Huy, Ngo Thu Hang, Can Van Mao, Tran Thi Thanh Huyen, Nguyen Linh Toan, Le Huu Song, C Thomas Bock, Heiner Wedemeyer, Thirumalaisamy P Velavan, Bui Tien Sy

**Affiliations:** Department of Pathophysiology, Vietnam Military Medical University, Hanoi, Vietnam; Department of Obstetrics and Gynaecology, 103 Military Hospital, Vietnam Military Medical University, Hanoi, Vietnam; Department of Pathophysiology, Vietnam Military Medical University, Hanoi, Vietnam; Institute of Biomedicine and Pharmacy, Vietnam Military Medical University, Hanoi, Vietnam; Vietnamese-German Center for Medical Research (VG-CARE), Hanoi, Vietnam; Vietnamese-German Center for Medical Research (VG-CARE), Hanoi, Vietnam; 108 Military Central Hospital, Hanoi, Vietnam; Institute of Biomedicine and Pharmacy, Vietnam Military Medical University, Hanoi, Vietnam; Institute of Tropical Medicine, University of Tübingen, Tübingen, Germany; Vietnamese-German Center for Medical Research (VG-CARE), Hanoi, Vietnam; 108 Military Central Hospital, Hanoi, Vietnam; Vietnamese-German Center for Medical Research (VG-CARE), Hanoi, Vietnam; 108 Military Central Hospital, Hanoi, Vietnam; 108 Military Central Hospital, Hanoi, Vietnam; Department of Pathophysiology, Vietnam Military Medical University, Hanoi, Vietnam; Department of Pathophysiology, Vietnam Military Medical University, Hanoi, Vietnam; Vietnamese-German Center for Medical Research (VG-CARE), Hanoi, Vietnam; 108 Military Central Hospital, Hanoi, Vietnam; Department of Pathophysiology, Vietnam Military Medical University, Hanoi, Vietnam; Vietnamese-German Center for Medical Research (VG-CARE), Hanoi, Vietnam; Vietnamese-German Center for Medical Research (VG-CARE), Hanoi, Vietnam; 108 Military Central Hospital, Hanoi, Vietnam; Infectious Diseases Departments, Robert Koch Institute, Berlin, Germany; Department of Gastroenterology, Hepatology, Infectious Diseases and Endocrinology at Hannover Medical School, Hannover, Germany; Vietnamese-German Center for Medical Research (VG-CARE), Hanoi, Vietnam; Institute of Tropical Medicine, University of Tübingen, Tübingen, Germany; Faculty of Medicine, Duy Tan University, Da Nang, Vietnam; Vietnamese-German Center for Medical Research (VG-CARE), Hanoi, Vietnam; 108 Military Central Hospital, Hanoi, Vietnam

**Keywords:** anti-HEV IgG, anti-HEV IgM, hepatitis B virus, hepatitis E virus, pregnancy

## Abstract

**Background:**

Hepatitis E virus (HEV) infection during pregnancy is associated with obstetric complications and adverse maternal and neonatal outcomes. This study aimed to determine the seroprevalence of HEV and RNA positivity in both healthy pregnant women and women coinfected with hepatitis B virus (HBV).

**Methods:**

A cross-sectional study was conducted involving 528 pregnant women (278 with and 250 without hepatitis B surface antigen [HBsAg]) in their third trimester. Anti-HEV specific immunoglobulin (Ig) G and IgM antibodies were tested for using enzyme-linked immunosorbent assay, while HEV RNA was detected by means of nested polymerase chain reaction. The status of anti-HEV antibodies was analyzed regarding pregnancy outcomes and the risks of obstetric complications.

**Results:**

The results indicated that 24% of participants (127 of 528) tested positive for anti-HEV IgG, while 2.5% (13 of 528) showed detectable anti-HEV IgM. Among HBV-positive women, 26% (55 of 250) had anti-HEV IgG, comparable to 22% (61 of 278) in HBV-negative controls. Notably, 28% (140 of 501) of cord blood samples were positive for anti-HEV IgG. No cases of HEV RNA were detected. The prevalence of anti-HEV IgG increased with maternal age and was associated with higher infant birth weights. Anti-HEV IgM positivity was associated with an increased risk of neonatal infections (odds ratio, 20.6; *P* = .05). Among HBsAg-positive women, those with anti-HEV IgG (26%) had higher gestational age at delivery and higher infant birth weights but lower platelet counts and prothrombin times (*P* < .05).

**Conclusions:**

These findings highlight the endemic nature of HEV in Vietnam and underscore the potential risks of coinfection with HBV during pregnancy, which may lead to adverse obstetric outcomes.

Hepatitis E virus (HEV) is a major cause of acute viral hepatitis, primarily transmitted through the fecal-oral route and via blood transfusions [1]. Each year, approximately 20 million HEV infections occur worldwide, leading to about 3.3 million symptomatic cases. In 2015, hepatitis E was responsible for approximately 44 000 deaths globally, accounting for 3.3% of all viral hepatitis-related deaths [2]. HEV is distributed worldwide, with some of the highest prevalence rates in Southeast Asia, including Vietnam [2]. In Vietnam, the seroprevalence of HEV among healthy individuals and blood donors has been reported at 31% and 26.8%, respectively [3, 4].

HEV is a single-stranded RNA virus belonging to the *Paslahepevirus* genus of the Hepeviridae family. Four genotypes mostly infect humans: HEV-1–HEV-4. HEV-1 and HEV-2 are predominantly transmitted through the fecal-oral route, typically in areas with poor sanitation. In contrast, HEV-3 and HEV-4 are zoonotic, transmitted through the consumption of undercooked meat from infected animals, by direct contact with animals, or via contaminated blood transfusions [2, 4]. In acute HEV infections, the incubation period ranges from 2 to 9 weeks. HEV RNA can be detectable in the blood 2–6 weeks after infection, with viral shedding in the feces lasting up to 2 weeks thereafter [1]. Immunoglobulin (Ig) M antibodies appear in the blood approximately 3–4 weeks after infection and persist for 4–6 months, while IgG antibodies emerge later and can last for several years after recovery [5].

Acute HEV infection typically resolves on its own, with a mortality rate of <4% in most populations. However, in pregnant women, the mortality rate can rise to 20% due to the risk of acute liver failure [6]. HEV infection has been associated with pregnancy complications such as ruptured amniotic membranes, spontaneous abortion, and stillbirth. Maternal complications have also been documented, including preeclampsia, hemorrhage, and acute liver failure. Some studies report an elevated risk of premature birth, miscarriage, maternal death, or fetal/neonatal death in pregnant women with HEV [7, 8]. Notably, HEV RNA has been detected in umbilical cord blood of about 50% of HEV RNA–positive pregnant women, underscoring the importance of screening umbilical cord blood for HEV during delivery [9].

Vietnam also has a high prevalence of hepatitis B virus (HBV) infection, with >8% of the population testing positive for hepatitis B surface antigen (HBsAg) [10]. Studies suggest that seroprevalence may be higher in individuals coinfected with HBV than in healthy individuals [4], but this association cannot be confirmed [11]. While acute HEV infection poses a significant risk during pregnancy and leads to an increased rate of maternal and neonatal complications, there are limited data on the prevalence and clinical profile of HEV in healthy pregnant women and in women coinfected with HBV, especially in countries with high HBV prevalence, such as Vietnam. This gap highlights the need for a targeted study to better understand the prevalence of HEV infection and its impact on maternal and fetal health in these populations. The current study aims to determine the prevalence of HEV infection in healthy pregnant women and those with positive HBsAg status, while also analyzing the clinical and subclinical characteristics of pregnant women with positive HEV antibodies.

## MATERIALS AND METHODS

### Ethical Statement

Written informed consent was obtained from all pregnant women during delivery. The study protocol was approved by the Ethics Committee of Military Hospital 103, Military Medical Academy, Hanoi, Vietnam (ethics approval no. 32/CNChT-HĐĐĐ). All recruitment procedures, sampling and experiments were conducted in accordance with ICH-GCP/GCLP guidelines and regulations.

### Participant Enrollment and Data Collection

The study used a cross-sectional descriptive sampling method to recruit 528 pregnant women between December 2021 and April 2024, divided into HBsAg-positive and HBsAg-negative groups, from hospitals across different regions in Vietnam. The pregnant women, all in their third trimester, were enrolled from hospitals across the Northern, Central Highlands, and Southern regions, including 250 HBsAg-positive women and 278 HBsAg-negative women, who served as controls. Consecutive eligible pregnant women were recruited from accessible hospitals using a consistent approach for both HBsAg-positive and HBsAg-negative groups, ensuring comparability between the 2 cohorts. The majority of HBsAg-positive participants had not received antiviral treatment for HBV. The sample size was determined based on an expected prevalence of 8% in the general population, with a 95% confidence level and a 5% margin of error [4]. The participants, with or without HBsAg-positive status, were selected using a cross-sectional approach, aiming to represent the population across multiple regions of Vietnam.

Peripheral and umbilical cord blood samples were collected at the time of delivery, serving as the testing time window. Serum was separated from 5 mL of peripheral blood and 5 mL of umbilical cord blood from all participating pregnant women. A strict cold chain was maintained throughout the process to ensure the integrity of the samples during transportation to the laboratory. Relevant patient data were collected from the medical records and clinical examination, including obstetric history (total pregnancies, miscarriages, stillbirths, and premature births), maternal age, amniotic fluid status at admission, and postpartum details on newborns, such as gestational age, birth weight, and delivery complications. HBV viremia quantification was not possible, as it is not part of the clinical routine in pregnant women with HBsAg-positive status. Clinical laboratory tests for the pregnant women included red blood cell count, hemoglobin level, platelet count, liver enzyme levels (aspartate aminotransferase [AST] and alanine aminotransferase [ALT]), and prothrombin time.

### Enzyme-Linked Immunosorbent Assay Screening for Anti-HEV IgG and IgM Antibodies

Peripheral blood serum samples were screened for anti-HEV IgG and IgM antibodies, while cord blood serum samples were screened only for anti-HEV IgG antibodies, using a commercial enzyme-linked immunosorbent assay (ELISA) kit (Wantai) according to the manufacturer's instructions. The ELISA plates were read at an optical density of 450 nm. Results were considered positive when the optical density result divided by the cutoff value was ≥1, and negative when it was ≤1. Samples showing results between 0.9 and 1.1 were repeated to confirm the initial findings.

### RNA Extraction and Nested Polymerase Chain Reaction for HEV Detection

RNA was extracted from 140 µL of serum samples using the QIAmp Viral RNA Mini Kit (Qiagen) according to the manufacturer's instructions. The quantity and quality of the RNA were assessed using a NanoDrop spectrophotometer. The RNA was then transcribed into complementary DNA (cDNA) using the LunaScript RT SuperMix Kit (New England BioLabs) and subsequently stored at −80°C. All samples were tested for HEV RNA using nested polymerase chain reaction (PCR) targeting the viral open reading frame (ORF) 1 and ORF2 regions, as described by Hoan et al [4, 12]. The nested PCR targeting both ORF-1 and ORF-2 followed the protocol described elsewhere [13]. For ORF1, the outer primer pairs were HEV-38 (sense) 5′-GAG GCY ATG GTS GAG AAR G-3′ and HEV-39 (antisense) 5′-GCC ATG TTC CAG ACR GTR TTC C-3′, while the inner primers were designated HEV-37 (sense) 5′-GGT TCC GYG CTA TTG ARA ARG-3′ and HEV-27 (anti-sense) 5′-TCR CCA GAG TGY TTC TTC C-3′. For ORF2, the outer primers were HEV-34 (sense) 5′-CCG ACG TCY GTY GAY ATG AA-3′ and HEV-36 (anti-sense) 5′-TTR TCC TGC TGA GCR TTC TC-3´, and the inner primers were HEV-35 (sense) 5′-AAG TGA GCG CCT ACA YTA YCG-3′ and HEV-29 (anti-sense) 5′-CTC GCC ATT GGC TGA GAC-3′.

PCR amplification was performed in a 25-µL reaction volume containing 50 ng of viral cDNA, 1× PCR buffer, 0.4-mmol/L deoxynucleotide triphosphates, 0.4-mol/L magnesium chloride, 0.6-µmol/L specific primer pairs, and 1 unit of Taq polymerase (Qiagen). The thermocycling parameters for the outer ORF1 PCR included an initial denaturation at 94°C for 5 minutes, followed by 30 cycles of denaturation (95°C for 30 seconds), annealing (56°C for 30 seconds), and extension (72°C for 40 seconds), concluding with a final extension at 72°C for 10 minutes. The thermocycling parameters for the inner ORF1 nested PCR were similar, with an initial denaturation at 94°C for 5 minutes, followed by 36 cycles of denaturation (95°C for 30 seconds), annealing (54°C for 30 seconds), and extension (72°C for 30 seconds), ending with a final extension at 72°C for 10 minutes. For ORF2, the thermal cycling program was the same as that for ORF1, with annealing temperatures of 54°C for PCR and 56°C for nested PCR. A plasmid containing HEV cDNA served as a positive control. The amplicons (307 base pairs [bp] for ORF1 and 489 bp for ORF2) were visualized on 1.1% agarose gels stained with SYBR Green. Samples positive for either ORF1 or ORF2 were considered positive for HEV RNA, and all positive samples were replicated for confirmation.

### Statistical Analyses

The prevalence rates and quantitative variables are presented as percentages, medians, and interquartile ranges. Categorical variables were tested using χ^2^ or Fisher exact tests, while quantitative variables were compared using *t* or Mann-Whitney tests. Multivariate analyses (linear and logistic regression models) were performed to assess the association between potential risk factors and adverse pregnancy outcomes. Odds ratios and 95% confidence intervals were calculated. All statistical analyses were performed using SPSS 25 software, with the significance level set at *P* < .05.

## RESULTS

### Demographic and Clinical Characteristics of Pregnant Women

The average age of the HBsAg-positive pregnant women was 29.9 years, significantly higher than the 28.55 years observed in the HBsAg-negative group (*P* = .003). The HBsAg-positive group also had a slightly higher rate of miscarriages (*P* = .054) compared with the HBsAg-negative group. Infants born to HBsAg-positive mothers had higher birth weights (*P* = .01). Clinical laboratory findings revealed lower white blood cell and platelet counts in the HBsAg-positive group (*P* < .05), while AST and ALT levels were significantly elevated (*P* < .001). No other significant differences were observed between the 2 groups (Table 1).

**Table 1.** Demographic and Clinical Characteristics of the Pregnant Women

Characteristic	Mean		P Value^a^
	HBsAg-Positive Women (n = 250)	HBsAg-Negative Women (n = 278)	
Maternal age, y	29.9 (5.1)	28.55 (5.2)	.003
Gestational age, wk	39 (0.9)	39 (1)	NS
Parity	2.2 (1.1)	2.1 (1)	NS
Total no. of deliveries	1.0 (0.9)	0.96 (0.9)	NS
No. of miscarriages	0.2 (0.5)	0.14 (0.4)	.054
Mode of delivery, no. (%)			
Cesarean	83 (33.2)	29 (10.4)	<.001^b^
Vaginal	167 (66.8)	249 (89.6)	
Amniotic condition, no. (%)			
Unruptured	223 (89.2)	234 (84.2)	NS
Ruptured	27 (10.8)	44 (15.8)	
Birth weight, g	3208.5 (334.5)	3137.7 (348.4)	.01
White blood cells, g/L	9.5 (2.3)	9.9 (2.4)	.03
Platelets, G/L	216.6 (55.6)	228.6 (61)	.02
ALT, U/L	19.6 (14.1)	13.7 (6.7)	<.001
AST, U/L	23.3 (11.7)	18.8 (4.3)	<.001
Prothrombin time, s	11.3 (1.1)	11 (0.6)	NS
APTT, s	27 (2.9)	26.4 (2.9)	NS

Abbreviations: ALT, alanine aminotransferase; APTT, activated partial thromboplastin time; AST, aspartate aminotransferase; G/L, giga per liter; NS, not significant; T/L, tera per liter.

^a^ *P* values based on Mann-Whitney test unless otherwise specified.

^b^Based on χ^2^ test.

### Seroprevalence of Anti-HEV Antibodies in Pregnant Women

ELISA results showed that 24% (127 of 528) pregnant women had detectable anti-HEV IgG and 2.5% (13 of 528) had detectable anti-HEV IgM antibodies. Among the HBsAg-positive women, 26% (66 of 250) were positive for anti-HEV IgG and 2.4% (6 of 250) for anti-HEV IgM. In the HBsAg-negative group, 22% (61 of 278) were positive for anti-HEV IgG and 2.5% (7 of 278) for anti-HEV IgM. No significant difference in anti-HEV IgM prevalence was observed between groups (*P* > .05) (Table 2). Of the total 528 participants, cord blood samples from 27 HBsAg carriers were unavailable. Overall, 28% of the cord blood samples (140 of 501) were positive for anti-HEV IgG. The IgG titers showed no significant differences between groups (*P* > .05). Among IgG-positive pregnant women, 92% were simultaneously positive in both peripheral and cord blood. Furthermore, 7.7% (9 of 117) had anti-HEV IgG in peripheral blood but not cord blood samples, while 4.7% (18 of 384) had IgG in cord blood but not peripheral blood samples (data not shown).

**Table 2.** Association of Anti–Hepatitis E Virus Antibodies With Clinical Parameters Among All Pregnant Women

Characteristic	Mean		P Value^a^
	Anti-HEV IgG or IgM Positive	Anti-HEV IgG or IgM Negative	
Anti-HEV IgG, no. (%)	127 (24.05)	401 (75.95)	…
Maternal age, y	31 (5.2)	28.6 (5.1)	<.001
Maternal age group, no. (%)			
≤35 y	100 (78.7)	365 (91)	<.001^b^
>35 y	27 (21.3)	36 (9)	
Gestational age, wk	39.2 (0.8)	38.9 (0.9)	.003
Amniotic condition, no. (%)			
Unruptured	112 (88.2)	345 (86)	NS
Ruptured	15 (11.8)	56 (14)	
Birth weight, g	3279.1 (351.1)	3137.1 (334.2)	<.001
Birth weight category, no. (%)			
<2800 g	13 (10.2)	68 (17)	.01^b^
2800–3499 g	81 (63.8)	269 (67.1)	
3500–3999 g	28 (22.1)	60 (15)	
>4000 g	5 (3.9)	4 (0.8)	
Parity	2.4 (1.1)	2.1 (1)	.001
Parity category, no. (%)			
1	26 (20.6)	138 (34.4)	.004^b^
>1	100 (79.4)	263 (65.6)	
Total no. of deliveries	1.2 (0.9)	0.9 (0.9)	.002
Total no. of deliveries, no. (%)			
1	30 (23.6)	154 (38.4)	.002^b^
>1	97 (76.4)	247 (61.6)	
No. of miscarriages	0.28 (0.6)	0.1 (0.4)	.02
Platelets, T/L	211.3 (55.5)	226 (59.3)	.01
AST, U/L	21.6 (9.8)	20.8 (8.8)	NS
ALT, U/L	16.7 (11.9)	16.6 (11.2)	NS
Prothrombin time, s	11 (0.8)	11.2 (0.9)	.01
APTT, s	26.8 (2.6)	26.6 (3)	NS
Anti-HEV IgM, no. (%)	13 (2.5)	515 (97.5)	…
Maternal age, y	29.5 (5.6)	29.2 ( 5.2)	NS
Gestational age, wk	38.7 (0.9)	39 (0.9)	NS
Amniotic condition, no. (%)			
Unruptured	12 (92.3)	455 (92.3)	NS
Ruptured	1 (7.7)	70 (7.7)	
Birth weight, g	3226.2 (306.6)	3169.8 ( 344.4)	NS
Parity	2 (0.8)	2.2 (1)	NS
Total no. of deliveries	0.9 (0.9)	1 (0.9)	NS
No. of miscarriages	0.15 (0.4)	0.18 (0.5)	NS
Platelets, T/L	238 (56.3)	222.5 (58.8)	NS
AST, U/L	19.8 (7.9)	21.0 (9.1)	NS
ALT, U/L	15.9 (12.5)	16.7 (11.3)	NS
Prothrombin time, s	11.3 (1.02)	11.2 (0.9)	NS
APTT, s	28.1 (2.6)	26.6 ( 2.9)	NS

Abbreviations: ALT, alanine aminotransferase; APTT, activated partial thromboplastin time; AST, aspartate aminotransferase; HEV, hepatitis E virus; Ig, immunoglobulin; NS, not significant; T/L, tera per liter.

^a^ *P* values based on Mann-Whitney test unless otherwise specified.

^b^Based on χ^2^ test.

### Detection of HEV RNA in Pregnant Women

All pregnant women were tested for HEV RNA in both peripheral and cord blood samples using nested PCR targeting the ORF1 (306-bp) and ORF2 (497-bp) regions, which revealed no samples positive for HEV RNA.

### Maternal Age, Clinical Outcomes, and Anti-HEV Antibodies

Anti-HEV IgG prevalence increased significantly with age, with a higher proportion of women >30 years of age testing positive (*P* < .001). Mothers with anti-HEV IgG were older and delivered later in gestation (*P* = .003). Multiple pregnancies and higher delivery counts were associated with an increased risk of HEV infection (*P* < .05). Newborns of mothers with anti-HEV IgG had significantly higher birth weights (*P* < .001), and women with anti-HEV IgG showed lower platelet counts and shorter prothrombin times than those without antibodies (both *P* = .01), though no differences in AST and ALT levels were observed. We also compared clinical parameters between groups positive and negative for anti-HEV IgM and found no significant clinical differences (Table 2).

Among HBsAg-positive women, those with anti-HEV IgG (26%) had higher gestational ages at delivery, infant birth weights, parity, and delivery counts but lower platelet counts and prothrombin times (*P* < .05). No significant differences were observed in clinical parameters by anti-HEV IgM status. Among HBsAg-negative women, those with anti-HEV IgG had higher maternal age and infant birth weights than those without anti-HEV IgG (*P* < .05) (Table 3). For cord blood, mothers with anti-HEV IgG had higher maternal age, gestational age, and infant birth weights, findings consistent with those of HBsAg carriers (*P* < .05; Table 4).

**Table 3.** Association of Anti–Hepatitis E Virus Antibodies with Clinical Parameters in Hepatitis B Surface Antigen–Positive and Negative Pregnant Women

Characteristic	Mean					
	HBsAg-Positive Women (n = 250)			HBsAg-Negative Women (n = 278)		
	Anti-HEV IgG or IgM Positive	Anti-HEV IgG or IgM Negative	*P* Value^a^	Anti-HEV IgG or IgM Positive	Anti-HEV IgG or IgM Negative	*P* Value^a^
Anti-HEV IgG	(n = 66)	(n = 184)	…	(n = 61)	(n = 217)	…
Maternal age, y	31.7 (5.2)	29.2 (5)	.001^b^	30.3 (5.1)	28.1 (5.1)	.008
Gestational age, wk	39.3 (0.9)	38.9 (0.9)	.005	39.2 (0.8)	38.9 (1)	NS
Birth weight, g	3334.3 (352.2)	3163.4 (316.9)	<.001^b^	3219.3 (342.7)	3114.8 (347.3)	.03
Parity	2.6 (1.1)	2.1 (1.0)	.006	2.3 (1.2)	2 (1)	NS
Total no. of deliveries	1.3 (0.9)	0.9 (0.8)	.006	1.1 (0.9)	0.9 (0.9)	NS
Platelets, G/L	201.4 (54.7)	222 (55)	.01^b^	222.1 (54.8)	230.4 (62.6)	NS
AST, U/L	23.8 (12.4)	23.1 (11.5)	NS	19.2 (4.3)	18.6 (4.3)	NS
ALT, U/L	20.0 (15.1)	19.5 (13.8)	NS	13 (4.6)	14 (7.2)	NS
Prothrombin time, s	11.0 (0.9)	11.4 (1.1)	.02	10.9 (0.6)	11.0 (0.6)	NS
APTT, s	26.7 (2.5)	27 (3.1)	NS	27.1 (2.7)	26.2 (2.9)	NS
Anti-HEV IgM	(n = 6)	(n = 244)	…	(n = 7)	(n = 271)	…
Maternal age, y	30.7 (6)	29.9 (5.1)	NS	28.4 (5.5)	28.6 (5.2)	.006
Gestational age, wk	39 (0.9)	39 (0.9)	NS	38.4 (0.8)	39 (0.97)	NS
Birth weight, g	3271.7 (355.6)	3207 (334.6)	NS	3187.1 (280.6)	3136.4 (350.3)	.03
Parity	2.2 (0.7)	2.2 (1.1)	NS	1.9 (0.9)	2.1 (1)	NS
Total no. of deliveries	1.2 (0.8)	1 (0.9)	NS	0.7 (1)	1 (0.9)	NS
Platelets, G/L	197.5 (44.4)	217 (559)	NS	272.7 (40.6)	227.4 (61.0)	.02
AST, U/L	23.1 (11.0)	23.3 (11.8)	NS	16.9 (1.9)	18.8 (4.4)	NS
ALT, U/L	20.8 (17.4)	19.6 (14.1)	NS	11.7 (3.6)	13.8 (6.8)	NS
Prothrombin time, s	11.6 (1.5)	11.3 (1.1)	NS	11.1 (0.5)	11.0 (0.7)	NS
APTT, s	27.8 (1.9)	26.9 (2.9)	NS	28.3 (3.3)	26.4 (2.9)	NS

Abbreviations: ALT, alanine aminotransferase; APTT, activated partial thromboplastin time; AST, aspartate aminotransferase; G/L, giga per liter; HEV, hepatitis E virus; Ig, immunoglobulin; NS, not significant.

^a^ *P* values based on Mann-Whitney test unless otherwise specified.

^b^Based on *t* test.

**Table 4.** Characteristics of Pregnant Women by Cord Blood Anti–Hepatitis E Virus Immunoglobulin G Antibody Levels

Characteristic	Mean								
	All Women (n = 501)			HBsAg-Positive Women (n = 223)			HBsAg-Negative Women (n = 278)		
	IgG Positive (n = 126)	IgG Negative (n = 375)	P Value^a^	IgG Positive (n = 62)	IgG Negative (n = 161)	P Value^a^	IgG Positive (n = 64)	IgG Negative (n = 214)	P Value^a^
Maternal age, y	30.5 (5.5)	28.6 (5)	.002	30.9 (5.5)	29.2 (5)	.03	30.2 (5.6)	28.1 (4.9)	.03^b^
Gestational age, wk	39.2 (0.9)	38.9 (1)	.007	39.3 (0.9)	38.9 (0.9)	.006	39.1 (0.9)	38.96 (1)	NS
Birth weight, g	3245.6 (357)	3139 (335.5)	.004	3276.3 (365.6)	3171.6 (319.7)	.04	3215.8 (348.8)	3114.4 (345.6)	.04^b^
Platelets, G/L	215.2 (55.6)	226.4 (59.7)	NS	206.1 (53.3)	222.7 (56)	NS^b^	224.1 (56.7)	229.9 (62.2)	NS
AST, U/L	21.1 (7.3)	20.6 (9.0)	NS	22.7 (8.9)	23.2 (12.1)	NS	19.3 (4.4)	18.6 (4.3)	NS
ALT, U/L	16.3 (10.3)	16.6 (11.5)	NS	19.5 (12.9)	19.7 (14.7)	NS	12.9 (4.6)	14 (7.2)	NS
Prothrombin time, s	11.0 (0.8)	11.3 (0.9)	.03	11.1 (0.9)	11.5 (1.2)	.03^b^	10.9 (0.7)	11 (0.6)	NS
APTT, s	26.8 (2.5)	26.6 (3.1)	NS	26.6 (2.4)	27.1 (3.2)	NS	27 (2.7)	26.3 (2.9)	NS

Abbreviations: ALT, alanine aminotransferase; APTT, activated partial thromboplastin time; AST, aspartate aminotransferase; G/L, giga per liter; Ig, immunoglobulin; NS, not significant.

^a^ *P* values based on t test unless otherwise specified.

^b^Based on Mann-Whitney test.

### HBV and HEV Coinfection and the Outcome of Pregnancy

Maternal age is significantly associated with higher infant birth weight, lower platelet counts, and higher AST levels (*P* < .05). Anti-HEV IgM positivity in peripheral blood was associated with shorter gestation (*P* < .05) and increased risk of neonatal infection (odds ratio, 20.6 [95% confidence interval, .95–448.95]; *P* = .05). Higher parity and total number of deliveries were associated with increased miscarriage risk (*P* < .001). HBV infection was independently associated with elevated AST and ALT levels (Table 5). Notably, among the 13 women with anti-HEV IgM, 1 (7.7%) had elevated liver enzymes (AST and ALT), 6 (46%) were HBsAg positive, 1 (7.7%) experienced premature rupture of membranes, 1 (7.7%) had neonatal respiratory failure with a concurrent neonatal infection, and 2 (15.4%) had infants with low birth weight (<2800 g).

**Table 5.** Multivariate Analysis of Factors Associated with Adverse Pregnancy Outcomes^a^

Risk Factor	Gestational Age		Birth Weight		Infant Respiratory Failure		Neonatal Infection		Miscarriage		Platelets		AST		ALT	
	Β Value	*P* Value	Β Value	*P* Value	OR (95% CI)	*P* Value	OR (95% CI)	*P* Value	Β Value	*P* Value	Β Value	*P* Value	Β Value	*P* Value	Β Value	*P* Value
Maternal age	.05	NS	.16	.004^b^	0.9 (.7–1.2)	NS	1.02 (.7–1.4)	NS	.003	NS	−.2	<.001^b^	.13	.03^b^	.9	NS
Parity	−.04	NS	.1	NS	NA	NS	1.6 (.3–8.4)	NS	2.1	<.001^b^	0	NS	.1	NS	.14	NS
Total no. of deliveries	.04	NS	−.5	NS	NA	NS	0.5 (.05–4.9)	NS	−1.8	<.001^b^	.02	NS	−.1	NS	−.2	.056
HBsAg positive	0	NS	.06	NS	NA	NS	NA	NS	.06	NS	−.07	NS	.2	<.001^b^	.2	<.001^b^
Anti-HEV IgG positive (peripheral blood)	.06	NS	.1	NS	2.56 (.03–218.6)	NS	1.4 (.01–147.3)	NS	.03	NS	−.05	NS	−.05	NS	−.03	NS
Anti-HEV IgM positive (peripheral blood)	−.09	.048^b^	.06	NS	11.6 (.6–237.3)	NS	20.6 (.95–448.95)	.05	.02	NS	.06	NS	−.001	NS	.01	NS
Anti-HEV IgG positive (cord blood)	.08	NS	.01	NS	2.1 (.03–135.5)	NS	3 (.04–242.2)	NS	.01	NS	−.01	NS	.03	NS	−.01	NS

Abbreviations: ALT, alanine aminotransferase; AST, aspartate aminotransferase; CI, confidence interval; HEV, hepatitis E virus; Ig, immunoglobulin; NS, not significant; OR, odds ratio.

^a^Gestational age was recorded in weeks, birth weight in grams, platelet count in giga per liter, AST and ALT in units per liter, and maternal age in years.

^b^Significant at *P* < .05.

## DISCUSSION

Pregnant women infected with HEV, especially in the third trimester, are at higher risk of obstetric complications and poor clinical outcomes [9, 14]. Currently, there are no large-scale studies in Vietnam focusing on HEV prevalence or clinical outcomes in infected pregnant women. The current study provides important insights into the seroprevalence of HEV among pregnant women in Vietnam and its potential implications, particularly regarding HBV coinfection. Given the high endemic rates of both HEV and HBV in Vietnam, understanding their effects on pregnancy outcomes is crucial.

Immunosuppression during pregnancy, combined with chronic HBV infection, increases the risk of HEV infection and adverse clinical outcomes [4, 15, 16]. Our findings reveal a notable prevalence of anti-HEV IgG antibodies (24%) and a lower prevalence of anti-HEV IgM (2.5%) among pregnant women, consistent with prior reports [3, 17, 18]. In addition, the prevalence of HEV infection aligns with studies indicating that HBV carriers are at a heightened risk for HEV infection, as higher rates of HEV infection have also been observed in individuals with preexisting liver conditions [4]. The observed incidence of anti-HEV IgM (2.5%) was higher than in earlier studies in pregnant women [19] and comparable to rates seen in asymptomatic HEV-infected individuals [3]. The majority of HEV-infected pregnant women in our study (79%) were <35 years old, with an average age higher than in other studies [19, 20]. Prevalence rates among HBsAg-positive and negative women were similar, indicating widespread HEV exposure irrespective of HBV status, which reinforces the endemic nature of HEV in Vietnam.

While chronic HEV infection during pregnancy has been documented [21], our study did not detect any cases of HEV RNA, suggesting no active virus in circulation. Moreover, this absence may result from the timing of sample collection not coinciding with the peak of acute infection or a lack of chronic cases in our cohort. Consequently, we could not identify circulating HEV genotypes, although Vietnam is known to harbor genotypes 1–4 [1]. Even without HEV RNA–positive cases, prior HEV exposure in pregnant women has been associated with adverse outcomes, including earlier deliveries and lower birth weights. These findings underscore the health risks of HEV exposure in Vietnam, particularly for vulnerable populations like pregnant women. While increased maternal age is correlated with higher parity, some studies have not found a direct relationship between age, number of pregnancies, and births [20].

Notably, the association between the presence of anti-HEV IgG antibodies in cord blood and subsequent delivery outcomes in this cohort remains unclear and warrants further investigation. HEV replication in the placenta and vertical transmission from mother to fetus are risk factors for severe histopathological damage during pregnancy [16, 22, 23]. Our study found that 28% of pregnant women with HBsAg had anti-HEV IgG in their cord blood, slightly higher than the 23% in those without HBsAg. Maternal IgG antibodies are actively transported across the placenta, providing newborns with immediate immunity after birth [24]. In addition, during vaginal delivery, newborns may be exposed to maternal feces, increasing the risk of HEV exposure. Thus, the presence of anti-HEV antibodies in umbilical cord blood can help protect newborns from HEV during the early postpartum period when their immune systems are still developing.

Coinfection with HEV and HBV increases the risk of obstetric complications and adverse perinatal outcomes compared with monoinfections [25]. HBV infection was associated with elevated liver enzyme levels (AST and ALT); however, no significant clinical differences were observed between HBV-infected women with or without HEV antibodies. Given the absence of definitive cases of acute HEV infection in the cohort, it is unsurprising that elevated liver enzyme levels specifically attributable to acute HEV infection were not detected. Further studies are needed to explore potential interactions between HBV and HEV infections, particularly in cases of chronic HEV infection. Further investigation is warranted to understand potential interactions between these 2 infections, particularly in chronic HEV cases.

The relationship between HEV exposure and miscarriage risk was another key finding. This association, while not directly associated with HEV infection, may reflect underlying maternal health factors that amplify the risks posed during pregnancy. Our results align with findings of studies suggesting that high parity can be a risk factor for pregnancy complications [21, 26]. The association between anti-HEV IgM and adverse pregnancy outcomes, such as shorter gestation, infant respiratory failure, and neonatal infections, highlights the significant risks HEV poses during pregnancy. These findings are consistent with those of earlier studies showing that HEV infection in pregnancy can lead to severe complications, including maternal death, preterm birth, and fetal death [16, 18, 27].

In our study, 10% of low-birth-weight infants were born to mothers with anti-HEV IgG, a lower proportion than for those without these antibodies. Conversely, the number of macrosomic infants (>4 kg) was higher among mothers with anti-HEV IgG. This observed association may be influenced by residual confounding factors, such as socioeconomic status, poverty, or malnutrition, which are also potentially linked to anti-HEV IgG positivity. In addition, several studies have reported an association between HBV infection in pregnant women and a higher rate of macrosomic infants [28–30]. While acute HEV may negatively affect neonatal outcomes, the impact of prior HEV exposure remains unclear. Pregnant women with anti-HEV IgG delivered later than others, without increased risks of premature rupture of membranes or miscarriage, consistent with previous studies that reported no consensus on these risks [1, 7, 19, 20].

Clinical parameters such as thrombocytopenia, reduced prothrombin time, and altered platelet counts were associated with the presence of anti-HEV IgG. However, a Chinese study found no differences in platelet count or prothrombin time but reported elevated ALT levels in women with anti-HEV antibodies [31]. Reported pregnancy outcomes in earlier studies include risks of premature rupture of membranes, preterm birth, hepatitis flare-ups, and jaundice [19, 22, 31]. In our study, premature rupture of membranes occurred in 15 women (11.8%) with anti-HEV IgG and 1 (8%) with anti-HEV IgM, consistent with previous findings [22]. Therefore, coinfection with HEV and HBV contributes to conditions such as premature rupture of membranes, elevated ALT and AST levels, decreased platelet count, and prothrombin time, which are associated with outcomes such as macrosomia, fetal distress, neonatal respiratory distress, and postpartum jaundice.

This study has several limitations. First, its cross-sectional nature limits the ability to establish causality between HEV infection and pregnancy outcomes. Longitudinal studies are needed to better understand the timing and progression of HEV infections during pregnancy, as well as larger, population-based studies to validate our findings and further explore the observed associations. Second, the absence of HEV RNA detection in serum samples may suggest that the study captured women after infection, missing active cases. Future studies should consider including stool samples, more sensitive methods, or larger cohorts to improve RNA detection rates. Finally, the lack of data on socioeconomic factors and dietary habits may limit context regarding HEV transmission and prevalence in this population.

In conclusion, HEV poses a significant public health concern for pregnant women in Vietnam, especially in the context of HBV coinfection. Anti-HEV IgM is associated with adverse pregnancy outcomes. Further research is needed to explore the interactions between HEV, HBV, and pregnancy outcomes and to develop targeted interventions to reduce the risk of HEV transmission in this vulnerable population.
